# Torsemide versus furosemide after acute decompensated heart failure: a retrospective observational study

**DOI:** 10.1186/s12872-019-1112-5

**Published:** 2019-05-28

**Authors:** Alaa Rahhal, Mohamed Omar Saad, Kawthar Tawengi, Abed Al Raouf Assi, Masa Habra, Dalia Ahmed

**Affiliations:** 1 Heart Hospital, Hamad Medical Corporation, P.O. Box 3050, Doha, Qatar; 2 Al-Wakra Hospital, Hamad Medical Corporation, Doha, Qatar

**Keywords:** Torsemide, Furosemide, Acute decompensated heart failure, Hospitalization

## Abstract

**Background:**

Loop diuretics are recommended by clinical practice guidelines to treat volume overload in acute decompensated heart failure (ADHF). The effectiveness of switching furosemide to torsemide versus optimizing the furosemide dose following ADHF has not yet been evaluated.

**Methods:**

This retrospective observational study aimed to assess the impact of switching furosemide to torsemide versus optimizing the furosemide dose after ADHF on HF-related hospitalization within 1 month and 6 months of discharge. The study included patients previously on furosemide admitted with ADHF to the Heart Hospital in Qatar between January 1, 2016 and June 30, 2017. The study included 2 groups: (1) patients discharged on torsemide; and (2) patients discharged on an optimized furosemide dose. Cox proportional hazard regression analysis was used to determine the association between diuretic use and hospitalization.

**Results:**

Of the 232 patients included, 45 received torsemide and 187 received an optimized furosemide dose upon discharge. The majority of patients included were males (54%) with a mean age of 67 ± 12 years, and presented with HF with reduced ejection fraction (57%) and had a history of coronary artery disease (68%). The 1-month and 6-month HF-related hospitalization did not differ between the torsemide and optimized furosemide groups (aHR = 0.72; 95% CI 0.23–2.3, *p* = 0.57; aHR = 0.94, 95% CI 0.45–1.8, *p* = 0.87), respectively.

**Conclusion:**

Switching furosemide to torsemide after ADHF was not associated with reduced HF-related hospitalization compared to receiving an optimized furosemide dose. Larger prospective clinical trials are needed to confirm the findings of this study.

## Background

Heart failure (HF) is considered the most common cause of hospital admissions in the United States leading to approximately 1.1 million hospitalizations annually [1]. Loop diuretics are recommended by clinical practice guidelines, including the latest American College of Cardiology Foundation/American Heart Association Heart Failure report ACCF/AHA (2013) (Class IB) and the European Society of Cardiology guidelines for the diagnosis and treatment of acute and chronic heart failure (2016) (Class IB), for treating volume overload and relieving symptoms of congestion among patients with acute decompensated heart failure (ADHF) with left ventricular dysfunction [2, 3].

Initially, patients presenting with acute decompensation of heart failure and already using furosemide should receive an intravenous furosemide dose that is at least equal to the oral home dose to treat fluid retention [2, 3]. Once fluid retention has resolved, patients will be discharged on oral diuretics to prevent the recurrence of volume overload [2]. Upon discharge, patients may receive higher doses of furosemide than their previous home doses to maintain euvolemia achieved with intravenous furosemide during the hospital stay, due to the low oral bioavailability of furosemide that is about 50% [4]. Torsemide, however, has increased bioavailability (> 80%) and a longer half-life compared with furosemide [4], promoting its use following ADHF in patients previously using furosemide or other diuretics [5]. Moreover, torsemide has protective effects on ventricular structure and beneficial effects on the neurohormonal axis [6]. Nevertheless, data guiding diuretic dosing after ADHF and the association with subsequent outcomes are limited. A number of clinical trials that compared torsemide versus furosemide in HF suggested improved morbidity and mortality with torsemide; however, these studies included patients with chronic heart failure without a recent hospitalization for ADHF and did not evaluate switching furosemide to torsemide versus optimizing the dose of furosemide upon discharge [7, 8]. Therefore, this retrospective observational study aims to evaluate the effectiveness of switching furosemide to torsemide versus increasing furosemide dose upon discharge among HF patients admitted with ADHF and to determine the predictors of switching furosemide to torsemide upon discharge.

## Methods

### Study setting

This study was conducted at the national Heart Hospital (HH) in Qatar. The HH is a 116-bed tertiary cardiology specialized hospital that is part of the Hamad Medical Corporation (HMC), the major healthcare provider in Qatar, and it is the only national center for cardiovascular diseases in Qatar [9].

### Study design and population

This retrospective observational study consisted of two phases: (1) determining the time to hospitalization for HF within 30-day and 6-month time periods following discharge among furosemide users who were shifted to torsemide compared to time to hospitalization for patients who received an optimized dose of furosemide; and (2) conducting a retrospective analysis of the demographics and clinical characteristics of the torsemide users and optimized furosemide users to identify the predictors of switching furosemide to torsemide following ADHF admission.

All HF patients, with reduced or preserved ejection fraction (EF), admitted with ADHF to the HH during the study period (January 1st, 2016 and June 30th, 2017) were screened and patients who were using furosemide prior to admission were identified. All furosemide users who were discharged on an optimized dose of furosemide, defined as a higher dose compared to the dose prior to the current admission, were included in the optimized furosemide arm (optimized furosemide users); and all furosemide users who were discharged on torsemide were include in the torsemide arm (torsemide users).

### Eligibility criteria

All HF patients, including patients with either reduced or preserved EF, already using furosemide and were admitted with ADHF and then discharged on either an optimized dose of furosemide or switched to torsemide were included.

### Follow-up and outcomes

The outcomes measured in this retrospective study were as follows: (1) Time to first hospitalization due to HF within 30 days and 6 months of discharge following switching furosemide to torsemide versus optimizing the dose of furosemide among HF patients admitted with ADHF. (2) Predictors of switching furosemide to torsemide among HF patients with prior use of furosemide admitted with ADHF, including several patient-related, disease-related, and medication-related factors: demographics, comorbid diseases, concomitant prescription drugs during hospitalization, and disease severity.

### Covariates

The results were adjusted for clinically relevant patient-, disease-, and medication-related variables that are associated with HF hospitalization: gender, age, EF, weight, baseline potassium, baseline creatinine clearance, baseline sodium, mitral regurgitation, mitral stenosis, aortic regurgitation, aortic stenosis, tricuspid regurgitation, cardiac resynchronization therapy defibrillator (CRTD), angiotensin converting enzyme (ACE) inhibitors, angiotensin II receptor blockers (ARBs), thiazide-likediuretics, beta-blockers, aldosterone antagonists, digoxin, intravenous diuretic use, and home diuretics dose.

To calculate the total daily diuretic dose upon discharge, torsemide diuretic doses were converted to furosemide-equivalents on the basis of 20 mg of torsemide is equivalent to 40 mg of furosemide [2, 3].

### Data collection procedures

Data were collected from the HMC electronic medical records using Cerner Electronic Medical Record System. Relevant data were manually extracted using a pretested data collection form. Outcomes of interest, including time to hospitalization for HF as well as patient-, disease-, and medication-related factors, were extracted accordingly.

### Statistical analyses

Statistical analyses were performed using the Statistical Package for Social Sciences program version 23.0 (IBM SPSS_ Statistics for Windows; IBM Corp, Armonk, NY). Descriptive statistics in the form of frequencies with percentages were reported for categorical variables, and means with standard deviations for continuous variables. The chi square test was used to compare categorical variables between the two groups (optimized furosemide vs. torsemide) and Student’s t-test was used to compare the means of continuous variables between the two groups. Two-way ANOVA was done to compare the mean change in diuretic dose from admission to discharge between the two groups.

Cox proportional hazard regression analyses were used to assess the association between torsemide use and time-to-hospitalization at 30 days and 6 months following discharge. The 30-day and 6-month Cox proportional hazard models were adjusted for clinically relevant variables. The results were presented as hazard ratio (HR), adjusted hazard ratio (aHR) with 95% confidence intervals (CIs). *P*-values of < 0.05 were used to indicate statistical significance.

Multivariate logistic regression was used to determine the predictors of switching furosemide to torsemide among HF patients. A total of clinically relevant variables were included in the logistic regression model, using the backward stepwise likelihood ratio with the probability of entry of 0.05 and removal of 0.10 at each step. The results are presented as crude odds ratio (OR), adjusted odds ratio (aOR) with 95% CI. *P*-values of < 0.05 were used to indicate statistical significance.

## Results

### Baseline characteristics

We identified 232 patients with HF who were already using furosemide and admitted with ADHF during the 18-month period (January 1st, 2016 to June 30th, 2017). Of the 232 patients identified, 45 patients were discharged on torsemide and 187 patients were discharged on an optimized dose of furosmide.

The patients included had a mean age of 67 ± 11.7 years, and more than half of the patients were males (54.3%) and had an EF of less than 40% (56.9%) as shown in Table 1.

**Table 1:** Baseline characteristics of heart failure patients previously on furosemide (*N* = 232)

Characteristic	All Patients (N = 232) n (%)	Torsemide Users (*N* = 45) n (%)	Optimized Furosemide Users (*N* = 187), n (%)	*P*-value
Male Gender	126 (54.3)	24 (53.3)	102 (54.5)	0.88
Age*	67 ± 11.7	65 ± 10.7	67 ± 11.9	0.23
Weight*	83 ± 23.9	88 ± 24.3	82 ± 23.7	0.15
Heart Failure				0.23
HFpEF	100 (43.1)	23 (51.1)	77 (41.2)	
HFrEF	132 (56.9)	22 (48.9)	110 (58.8)	
Ejection Fraction				0.751^§^
< 20%	8 (3.4)	2 (4.4)	6 (3.2)	
20–25%	38 (16.4)	10 (22.2)	28 (15.0)	
25–30%	32 (13.8)	4 (8.9)	28 (15.0)	
30–35%	32 (13.8)	3 (6.7)	29 (15.5)	
35–40%	22 (9.5)	3 (6.7)	19 (10.2)	
40–45%	25 (10.8)	7 (15.6)	18 (9.6)	
45–50%	30 (12.9)	5 (11.1)	25 (13.3)	
> 50%	45 (19.4)	11 (24.4)	34 (18.2)	
Systolic Blood Pressure*	119 ± 18.2	120 ± 17.6	119 ± 18.3	0.92
Diastolic Blood Pressure*	67 ± 9.1	68 ± 9.5	67 ± 9.0	0.41
Heart Rate*	74 ± 12.8	74 ± 12.6	74 ± 12.9	0.87
Serum Potassium*	4.2 ± 0.46	4.15 ± 0.4	4.19 ± 0.47	0.55
Serum Creatinine*	137 ± 82.8	144 ± 64.3	135 ± 86.7	0.51
Creatinine Clearance*	61 ± 31.9	60 ± 27.9	61 ± 32.8	0.75
Serum Sodium*	137 ± 4.4	137 ± 4.8	137 ± 4.3	0.82
Baseline Furosemide (mg)*	66 ± 39	101 ± 47	57 ± 31	< 0.001
IV Furosemide	222 (95.7)	40 (88.9)	182 (97.3)	0.026^‡^
Concurrent Medical Conditions				
Atrial Fibrillation	70 (30.2)	17 (37.8)	53 (28.3)	0.22
Coronary Artery Disease	158 (68.1)	24 (53.3)	134 (71.7)	0.018
Hypertension	199 (85.8)	39 (86.7)	160 (85.6)	0.85
Dyslipidemia	57 (24.6)	9 (20.0)	48 (25.7)	0.43
Diabetes Mellitus	185 (79.7)	34 (75.6)	151 (80.7)	0.44
Chronic Kidney Disease	99 (42.7)	22 (48.9)	77 (41.2)	0.35
Mitral Regurgitation	46 (19.8)	12 (26.7)	34 (18.2)	0.2
Mitral Stenosis	4 (1.7)	0 (0.0)	4 (2.1)	0.1^‡^
Aortic Regurgitation	4 (1.7)	1 (2.2)	3 (1.6)	0.58^‡^
Aortic Stenosis	14 (6)	4 (8.9)	10 (5.3)	0.48^‡^
Tricuspid Regurgitation	29 (12.5)	8 (17.8)	21 (11.2)	0.23
CABG	39 (16.8)	6 (13.3)	33 (17.6)	0.49
PCI	82 (35.3)	12 (26.7)	70 (37.4)	0.18
ICD	25 (10.8)	7 (15.6)	18 (9.6)	0.28^‡^
CRTD	14 (6.0)	6 (13.3)	8 (4.3)	0.03^‡^
Pacemaker	18 (7.8)	5 (11.1)	13 (7.0)	0.36^‡^

*Values expressed as mean ± SD; ^‡^*P*-value obtained through Fisher’s Exact test; ^§^*P*-value obtained through Mann-Whitney test; *HFpEF* heart failure with preserved EF, *HFrEF* heart failure with reduced EF, *CABG* coronary artery bypass grafting, *PCI* percutaneous coronary intervention, *ICD* implantable cardioverter defibrillator, *CRTD* cardiac resynchronization therapy defibrillator

Approximately 70% of patients treated with optimized doses of furosemide had coronary artery disease with 17.6% having a history of coronary artery bypass grafting (CABG) and 37.4% having a history of percutaneous coronary intervention (PCI). On the other hand, those switched to torsemide had higher prevalence of different valvular diseases, including mitral regurgitation, aortic regurgitation, aortic stenosis, and tricuspid regurgitation, except for mitral stenosis which was higher among the patients who received optimized furosemide doses (2.1% vs. 0%) as shown in Table 1. Similarly, patients switched from furosemide to torsemide following ADHF, compared to patients on an optimized furosemide dose had more ICD and CRTD implanted, (15.6% versus 9.5 and 13.3% versus 4.3%, respectively).

The baseline total daily dose of furosemide was significantly higher in the torsemide arm compared to the optimized furosemide arm (101 ± 47 mg per day versus 57 ± 31 mg per day, *p* < 0.001). Total daily doses of diuretics at discharge, expressed as furosemide-equivalent, were not statistically significant between the torsemide arm and the optimized furosemide arm (96 ± 54 mg versus 110 ± 46 mg, *p* = 068). Thus, the mean change in diuretic dose from admission to discharge was significantly lower in the torsemide arm (− 4.44 mg) versus that in the optimized furosemide arm (+ 53.69 mg), *p* < 0.001).

### Concurrent heart failure medications

Compared to patients in the optimized furosemide arm, almost double the patients in the torsemide arm were prescribed thiazide-like diuretics (15.6% vs. 7.0%, *p* = 0.08). Additionally, more patients in the torsemide arm were on aldosterone antagonists (44.4% vs. 34.2%) as shown in Table 2.

**Table 2:** Concurrent medications (N = 232)

Medication Class	All Patients (N = 232) n (%)	Torsemide Users (N = 45) n (%)	Optimized Furosemide Users (N = 187), n (%)	*P*-value
ACE Inhibitor	81 (34.9)	12 (26.7)	69 (36.9)	0.2
ABR	56 (24.1)	8 (17.8)	48 (25.7)	0.27
Thiazide Diuretic	6 (2.6)	1 (2.2)	5 (2.7)	1.0^‡^
Thiazide-Like Diuretic	20 (8.6)	7 (15.6)	13 (7.0)	0.08^‡^
Beta-Blocker	207 (89.2)	37 (82.2)	170 (90.9)	0.11^‡^
Aldosterone Antagonist	84 (36.2)	20 (44.4)	64 (34.2)	0.2
Dihydropyridine CCB	65 (28.0)	10 (22.2)	55 (29.4)	0.34
Non-Dihydropyridine CCB	6 (2.6)	1 (2.2)	5 (2.7)	1.0^‡^
Hydralazine	63 (27.2)	13 (28.9)	50 (26.7)	0.8
Nitrate	102 (44.0)	18 (40.0)	84 (44.9)	0.55
Digoxin	28 (12.1)	4 (8.9)	24 (12.8)	0.47
Ivabradine	6 (2.6)	0 (0.0)	6 (3.2)	0.6^‡^
Sacubitril/Valsartan	4 (1.7)	1 (2.2)	3 (1.6)	0.58^‡^
Diuretic TDD (mg)	108 ± 48	96 ± 54	110 ± 46	0.07

‡*P*-value obtained through Fisher’s Exact test, *ACE* angiotensin converting enzyme, *ARB* angiotensin II receptor blocker, *CCB* calcium channel blocker, *TDD* total daily dose

More patients in the optimized furosemide arm than the torsemide arm were using beta-blockers (90.9% vs. 82.2%, *p* = 0.11) and digoxin (12.8% vs. 8.9%, *p* = 0.47). Moreover, a higher percentage of patients in the optimized furosemide arm than the torsemide arm were on ACE inhibitors (36.9% vs. 26.2%, *p* = 0.2) and ARBs (25.7% vs. 17.8%, *p* = 0.27).

### Heart failure hospitalization

**Fig. 1:**
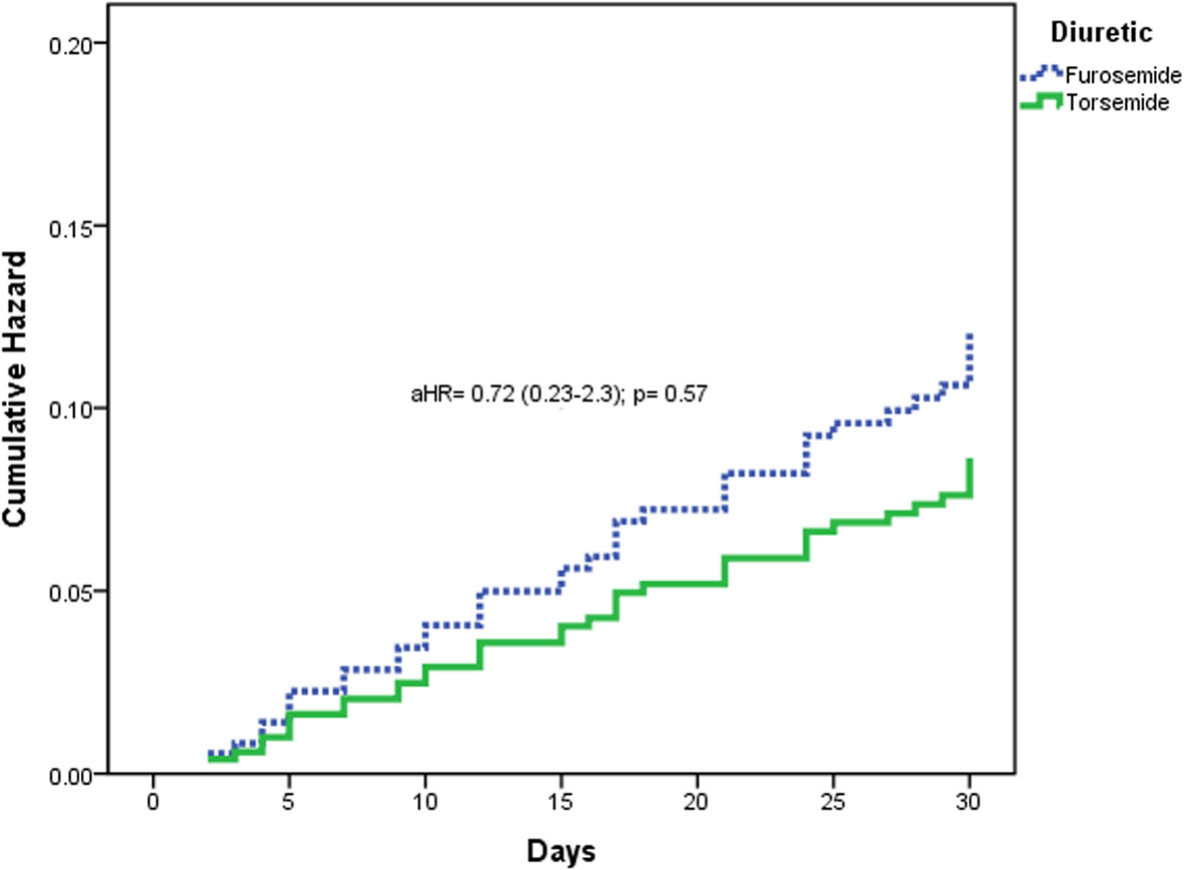
Kaplan-Meier adjusted 30-day hospitalization

Within 1 month of discharge after ADHF, torsemide use resulted in less HF hospitalization (15.6% vs. 16.6%, HR = 0.89, 95 CI 0.39–2.0, *p* = 0.77). After adjusting for variables that are associated with HF hospitalization: gender, age, EF, weight, potassium, creatinine clearance, sodium, valvular diseases, CRTD implantation, ACE inhibitors, ARBs, thiazide-like diuretics, beta-blockers, aldosterone antagonists, digoxin, intravenous diuretic use, and home diuretics dose, the HF hospitalization within 1 month did not differ significantly between the two groups (aHR = 0.72, 95% CI 0.23–2.3, *p* = 0.57) as demonstrated in Fig. 1.

**Fig. 2:**
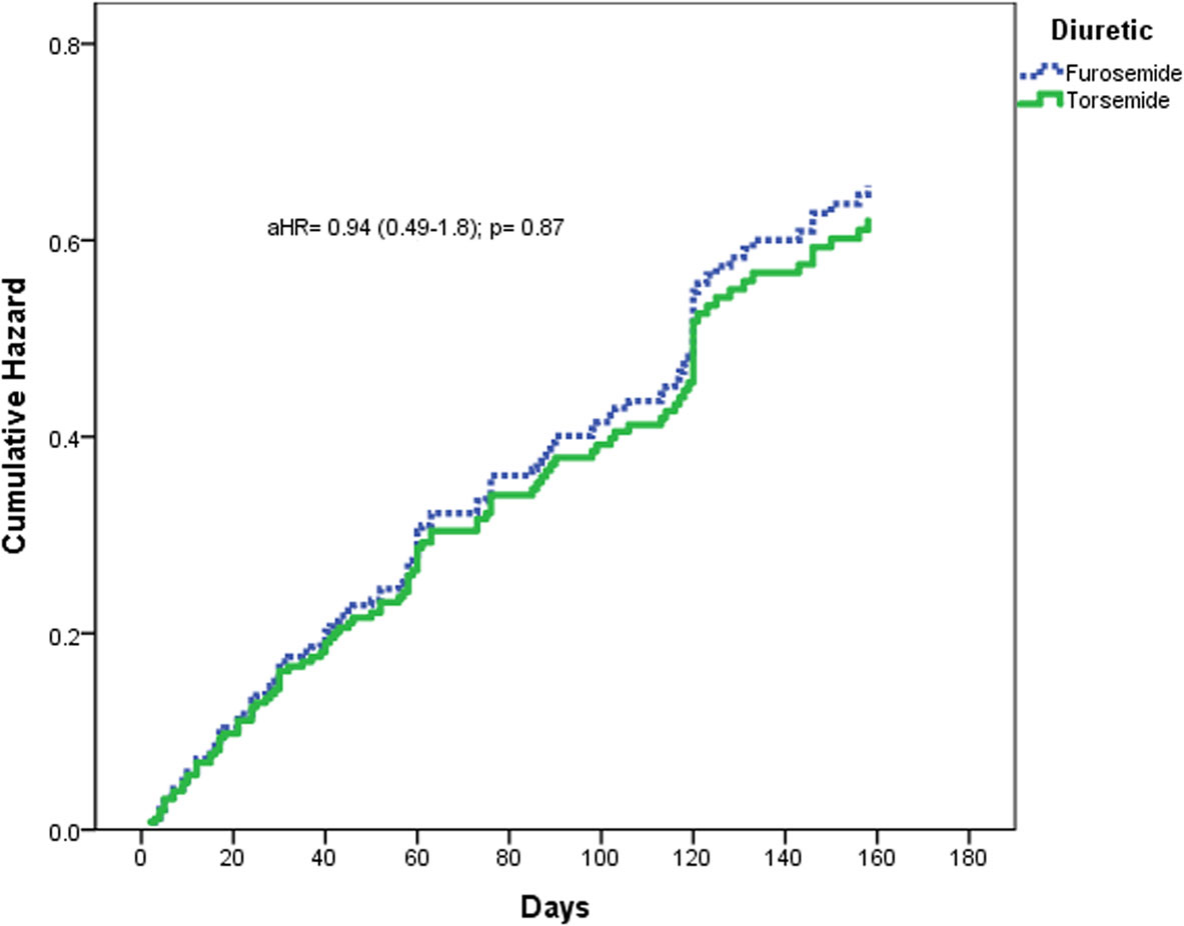
Kaplan-Meier adjusted 180-day hospitalization

Similarly, HF hospitalization within 6 months of discharge post ADHF was not significantly different between the two arms (torsemide 49.9% vs. optimized furosemide 46.0%, HR = 0.99, 95% CI 0.62–1.6, *p* = 0.98, aHR = 0.94, 95% CI 0.49–1.8, *p* = 0.87) as shown in Table 3 and Fig. 2.

**Table 3:** Outcomes of torsemide use

Outcome	Torsemide (N = 45) n (%)	Furosemide (N = 187) n (%)	Hazard Ratio 95% CI	*P*-value	Adjusted Hazard Ratio 95% CI	*P*-value
Hospitalization for HF within 1 month	7 (15.6)	31 (16.6)	0.89 (0.39–2.0)	0.77	0.72 (0.23–2.3)	0.57
Hospitalization for HF within 6 months	22 (48.9)	86 (46)	0.99 (0.62–1.6)	0.98	0.94 (0.49–1.8)	0.87

*HF* heart failure

### Predictors of Torsemide use

As shown in Table 4, the use of aldosterone antagonists increased the likelihood of prescribing torsemide among HF patients by almost 3 times (aOR = 2.7, 95% CI 1.1–6.7, *p* = 0.033), and higher EF increased the likelihood of using torsemide (aOR for each 5% increase in EF = 1.2, 95% CI 1.0–1.5, *p* = 0.046). On the other hand, age (aOR = 0.97, 95% CI 0.94–1.0, *p* = 0.065) and the use of either ACE inhibitors or ARBs (aOR = 0.4, 95% CI 0.2–0.94, *p* = 0.034) were negative predictors of torsemide use.

**Table 4:** Predictors of torsemide use

Characteristic	Adjusted Odds Ratio 95% CI	*P*-value
Age	0.97 (0.94–1.0)	0.065
Aldosterone Antagonist	2.7 (1.1–6.7)	0.033
ACE Inhibitor or ABR use	0.4 (0.2–0.94)	0.034
EF (5% increase)	1.2 (1.0–1.5)	0.046

*ACE* angiotensin converting enzyme, *ARB* angiotensin II receptor blocker, *EF* ejection fraction

## Discussion

In this retrospective observational study, we found that switching from furosemide to the more potent diuretic torsemide, compared to optimizing the dose of furosemide, following ADHF did not reduce the hospitalization due to HF within 1 month or 6 months of discharge. Since torsemide is a more potent loop diuretic with higher bioavailability and less erratic absorption in patients with HF that retains its pharmacodynamic effects regardless of the HF severity compared to furosemide [4, 5, 10], it was hypothesized that changing furosemide to torsemide would result in more favorable clinical outcomes than increasing the dose of furosemide following ADHF among patients already using furosemide prior to admission.

A systematic review and a meta-analysis of two randomized clinical trials that compared torsemide to furosemide in HF suggested that torsemide improved HF hospitalization (relative risk [RR] = 0.41, 95% CI 0.28–0.61, *p* < 0.0001) and cardiovascular mortality (RR = 0.86 95% Cl 0.53–1.39, *p* = 0.54); however, the two trials included in the analysis involved patients with chronic heart failure rather than ADHF [11]. Moreover, a large retrospective study (*n* = 4580) conducted at a tertiary center assessed the clinical outcomes, including hospitalization and mortality following admission for HF showed that torsemide use compared to furosemide was associated with significant increase in 30-day HF hospitalization (OR = 1.52, 95% CI 1.11–2.09, *p* = 0.0099); however, after adjustment for age, gender, chronic kidney disease, creatinine, ejection fraction, right ventricular size, aortic stenosis, mitral stenosis, tricuspid regurgitation, aldosterone antagonists, and ACE-inhibitors, torsemide use was not associated with increased HF hospitalization (aOR = 1.29, 95 CI 0.91–1.83, *p* = 0.1607). Nevertheless, the study neither evaluated changing furosemide to torsemide nor conducted an analysis of the patients already using a loop diuretic prior to admission [12].

Despite clinical and pharmacokinetic evidence favoring torsemide over furosemide in HF, our study did not demonstrate reductions in HF hospitalization at 1 or at 6 months with torsemide. However, our study answered a crucial clinical question of whether switching furosemide to a more potent diuretic (torsemide) following ADHF without increasing the dose would reduce HF-related hospitalization compared to increasing the furosemide dose. In our study, patients in the furosemide arm were discharged on double the baseline dose, while patients in torsemide arm were switched to torsemide at doses equivalent to the baseline furosemide dose and there was no difference between the two approaches in terms of clinical outcomes. Therefore, switching furosemide to an equivalent dose of torsemide may be as effective as optimizing the dose of furosemide after ADHF, which may represent a therapeutic advantage for torsemide.

In the present study, we investigated the predictors of torsemide use. Although these associations do not necessarily indicate causality, they might reflect a trend in prescribing pattern or highlight some indicators of disease progression to a stage where furosemide does not achieve the desired level of euvolemia. Interestingly, similar to Mentz et al., our study identified the use of aldosterone antagonists and higher EF as positive predictors of torsemide use in HF (OR = 1.97, 95% CI 1.59–2.44, *p* = < 0.0001; OR = 1.10, 95% CI = 1.07–1.14, *p* = < 0.0001; respectively), and increased age along with ACE inhibitor use as negative predictors of torsemide use (OR = 0.92, 95% CI 0.89–0.96, *p* = < 0.0001; OR = 0.78, 95% Cl 0.63–0.95, *p* = 0.015; respectively) [12].

This was a retrospective observational study which is susceptible to potential limitations. First, data were collected from the medical records with the expectation of missing some essential clinical information. Second, the study did not evaluate the impact on mortality of switching torsemide to furosemide versus optimizing the furosemide dose. However, we did not expect a difference in mortality due to lack of difference in placebo-controlled studies of furosemide. Third, the results were adjusted for clinically significant variables; however, there is a potential for other measured or unmeasured variables to influence the results. Forth, the number of torsemide users and optimized furosemide users were relatively small, which might have affected the robustness of the results. Nevertheless, this retrospective observational study aimed to answer a clinically important and challenging question that is faced by clinicians in daily practice, and it could serve as a preliminary indicator for future prospective studies to assess the impact of torsemide versus furosemide following ADHF on cardiovascular outcomes.

## Conclusions

In conclusion, switching furosemide to an equivalent dose of torsemide after ADHF was not associated with reduced HF-related hospitalization compared to optimizing the furosemide dose. Therefore, following ADHF, clinicians can follow either approach. However, larger prospective clinical trials are needed to confirm the findings of this study and to assess other important cardiovascular outcomes, including mortality.

## Data Availability

The datasets generated during this study are available from Hamad Medical Corporation electronic database, but restrictions apply to the availability of the data according to legal regulations of Qatar.

## Abbreviations

ACE: Angiotensin converting enzyme
ADHF: Acute decompensated heart failure
ARB: Angiotensin II receptor blockers
CABG: Coronary artery bypass grafting
CRTD: Cardiac resynchronization therapy defibrillator
EF: Ejection fraction
HF: Heart failure
HFpEF: Heart failure with preserved ejection fraction
HFrEF: Heart failure with reduced ejection fraction
ICD: Implantable cardioverter defibrillator
PCI: Percutaneous coronary intervention

## References

[1] Roger VL, Go AS, Lloyd-Jones DM (2011). Heart disease and stroke statistics--2011 update: a report from the American Heart Association. Circulation..

[2] Yancy CW, Jessup M, Bozkurt B (2013). 2013 ACCF/AHA guideline for the management of heart failure: a report of the American College of Cardiology Foundation/American Heart Association task force on practice guidelines. Circulation..

[3] Ponikowski P, Voors AA, Anker SD (2016). 2016 ESC guidelines for the diagnosis and treatment of acute and chronic heart failure. Eur Heart J.

[4] Felker GM, Mentz RJ (2012). Diuretics and ultrafiltration in acute decompensated heart failure. J Am Coll Cardiol.

[5] Heart Failure Society of America (2010). Executive summary: HFSA 2010 comprehensive heart failure practice guideline. J Card Fail.

[6] Harada K, Izawa H, Nishizawa T (2009). Beneficial effects of Torasemide on Systolic Wall stress and sympathetic nervous activity in asymptomatic or mildly symptomatic patients with heart failure: comparison with Azosemide. J Cardiovasc Pharmacol.

[7] Murray Michael D, Deer Melissa M, Ferguson Jeffrey A, Dexter Paul R, Bennett Susan J, Perkins Susan M, Smith Faye E, Lane Kathleen A, Adams Laurie D, Tierney William M, Brater D.Craig (2001). Open-label randomized trial of torsemide compared with furosemide therapy for patients with heart failure. The American Journal of Medicine.

[8] Cosín J, Díez J (2002). Torasemide in chronic heart failure: results of the TORIC study. Eur J Heart Fail.

[9] Aljundi AHS, Mohammed SFK, Patel A (2016). Inotropic agents use in patients hospitalized with acute decompensated heart failure: a retrospective analysis from a 22-year registry in a middle-eastern country (1991-2013). BMC Cardiovasc Disord.

[10] Bleske Barry E., Welage Lynda S., Kramer William G., Nicklas John M. (1998). Pharmacokinetics of Torsemide in Patients with Decompensated and Compensated Congestive Heart Failure. The Journal of Clinical Pharmacology.

[11] DiNicolantonio JJ (2012). Should torsemide be the loop diuretic of choice in systolic heart failure?. Futur Cardiol.

[12] Mentz RJ, Buggey J, Fiuzat M (2015). Torsemide Versus Furosemide in Heart Failure Patients. J Cardiovasc Pharmacol.

